# Self-assembly of gold nanoparticles to silver microspheres as highly efficient 3D SERS substrates

**DOI:** 10.1186/1556-276X-8-168

**Published:** 2013-04-12

**Authors:** Shouhui Chen, Peng Huang, Zhihua Wang, Zhe Wang, Magdalena Swierczewska, Gang Niu, Daxiang Cui, Xiaoyuan Chen

**Affiliations:** 1 Department of Bio-Nano-Science and Engineering, National Key Laboratory of Nano/Micro Fabrication Technology, Key Laboratory for Thin Film and Microfabrication of Ministry of Education, Institute of Micro-Nano Science and Technology, Shanghai JiaoTong University, Shanghai, 200240, China; 2 Laboratory of Molecular Imaging and Nanomedicine (LOMIN), National Institute of Biomedical Imaging and Bioengineering (NIBIB), National Institutes of Health (NIH), Bethesda, MD, 20892, USA

**Keywords:** Self-assembly, Gold nanoparticles, Silver microspheres, SERS

## Abstract

Herein we report a simple, one-pot, surfactant-free synthesis of 3D Ag microspheres (AgMSs) in aqueous phase at room temperature. The 3D AgMSs act as supports to fix the gold nanoparticles (GNPs) in 3D space via the interaction between the carboxyl groups of GNPs and the Ag atoms of AgMSs. The ensemble of AgMSs@GNPs with high surface-enhanced Raman scattering (SERS) activity and sensitivity can be an ideal 3D substrate choice for practical SERS detection applications. The simple self-assembly strategy may be extended to other metallic materials with great potentials in SERS, catalysis, and photoelectronic devices.

## Background

Ensembles of inorganic nanoparticles, which display unique collective properties that are different from those of both the individual nanoparticles and bulk materials, are of much scientific and technological interest [1-5]. The ensembles have the following potential advantages: (1) to display new electronic, magnetic, and optical properties as a result of interactions between the excitons, magnetic moments, or surface plasmons of individual nanoparticles; (2) to improve the mechanical properties of composite materials; and (3) to allow multiple tasks to be performed simultaneously or in sequence. Typically, the self-assembly of noble-metal nanoparticles has attracted much attention because of their unique plasmon resonance and their tremendous applications in the area of optical waveguides [6], superlensing [7], photon detection [8], and surface-enhanced Raman scattering (SERS) [9-12]. Recently, the SERS effect based on noble-metal ensembles is of particular interest because of its extraordinary ability to detect a wide variety of chemical/biological species at extremely low concentrations even down to the single-molecule level [9].

Gold nanoparticles (GNPs) have been widely used as Raman active substrates because of their good biocompatibility and strong SERS enhancement [13-18]. However, it should be mentioned that the particles tend to aggregate during aging, which results in an unwanted reduction of the active surface area [19,20]. To address this issue, the fixation of GNPs in one-dimensional (1D), 2D, or 3D spaces can avoid the aggregation of the particles as SERS substrates. Tsukruk et al. assembled GNPs onto 1D silver nanowires and 2D silver nanoplates to create bimetallic nanostructures as efficient single-nanoparticle Raman markers [21]. Li et al. developed a 2D GNP monolayer film as SERS substrate by the self-assembly of nanoparticles at a liquid/liquid interface [22]. Zhang et al. reported that GNPs dispersed on the grapheme oxide (GO, 2D) and reduced graphene oxide (RGO, 2D) supports exhibit excellent SERS and catalytic performance compared with the metal nanoparticles alone [23]. Qian et al. prepared the self-assembled 3D-ordered GNP precursor composite (SiO_2_/GNPs) arrays as SERS nanoprobes [24]. Choi et al. reported a highly ordered SERS-active surface that is provided by a 3D GNP array based on thermal evaporation of gold onto an indium tin oxide (ITO) surface through a nanoporous alumina mask [25]. This SERS-active surface was applied to analyze the intracellular state. Therefore, the development of appropriate support materials to fix GNPs is very important in practical SERS detection applications.

Recently, 3D Ag microspheres (AgMSs), which contain special fine structure, large specific surface area, and micron-sized particles, have been applied as SERS substrates [19,26]. For example, Zhao et al. prepared 3D AgMSs with nanotextured surface morphology by a simple, sonochemical, surfactant-free method. Due to their special structural features with nanoscale corrugations, the obtained 3D silver microstructures showed a structurally enhanced SERS performance [19]. Zhang et al. developed hierarchical assemblies of silver nanostructures as highly sensitive SERS platforms by an acid-directed assembly method [26]. Our group also used proteins [27] and microorganisms [28] as templates to synthesize AgMSs and hollow porous AgMSs, respectively. However, the controlled synthesis of AgMSs with clean rough surface is still a significant challenge.

**Figure 1:**
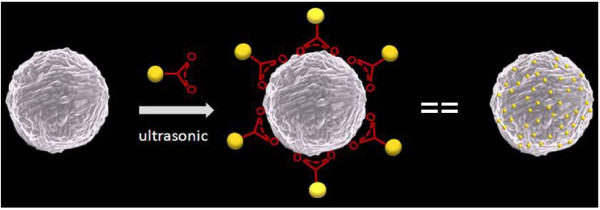
Schematic representation of the self-assembly between gold nanoparticles (GNPs) and Ag microspheres (AgMSs) via the coupling between the carboxyl groups of GNPs and the silver atoms of AgMSs.

Herein, we report a simple, ‘one-pot’, surfactant-free synthesis of 3D Ag microspheres by using silver nitrate (AgNO_3_) along with l-ascorbic acid (l-AA) as a reducer in aqueous phase at room temperature. The present method provides a facile and rapid route to the large-scale synthesis of 3D AgMSs with nanotextured surface morphology. The GNPs were successfully assembled on the clean rough surface of AgMSs via the interaction between the carboxyl groups of GNPs and the silver atoms of AgMSs (Figure 1).

## Methods

### Experimental section

#### Preparation of gold nanoparticles

Briefly, 50 mL (0.2 mg/mL) of chloroauric acid (Sigma-Aldrich) was heated to boiling point, and then 1.2 mL (10 mg/mL) of sodium citrate (Sigma-Aldrich) was added. Boiling lasted for 5 min until the solution became dark red in color. After cooling down to room temperature, 20 μL of GNPs was used for the analysis using transmission electron microscopy (TEM).

#### Zeta potential of the assemblies prepared at different molar ratios of Ag microspheres to gold nanoparticles

Typically, 2.5 mL of 5 mM AgNO_3_ aqueous solution was added to 95 mL of deionized (DI) water in a 150-mL beaker. Then, 2.5 mL of 5 mM l-AA (Sigma-Aldrich) was added into the above-mentioned solution under vigorous stirring at room temperature. The system was stirred vigorously under ambient conditions for 4 h. The color of the solution rapidly changed from colorless to gray. The resulting product was collected by centrifugation, washed three times with DI water and ethanol, and then dispersed in ethanol for further use.

#### Preparation of the assemblies of GNPs to AgMSs

AgMSs (10.8 mg) was dispersed in 0.9 mL of ethanol solution, then 100 μL of different concentrations of GNPs (0.4, 0.2, 0.1, 0.02, and 0.01 mg) were mixed with AgMSs solution under ultrasonic interaction, respectively. After 10 min, the resulting product was collected by centrifugation at 1,000 rpm for 5 min and washed twice with DI water and then dispersed in 1 mL DI H_2_O for further use.

#### Preparation of Raman samples

A total of 200 μL of GNPs to AgMSs (AgMSs@GNPs) was immersed in ethanol solutions containing 200 μL of 2-mercaptopyridine (2-Mpy) (10 to 7 M) under ultrasound for 10 min. After 2-Mpy molecules (Sigma-Aldrich) were adsorbed on the AgMSs@GNPs, the samples were washed twice with DI water and ethanol by centrifugation and finally dispersed in 10 μL ethanol. Then, an aliquot of 10 μL of 2-Mpy-loaded AgMSs@GNPs in ethanol solution was dropped onto a Si wafer. The dropped solution was spread evenly into a circle. After evaporation of ethanol under the dry N2, the sample was measured by a simple Raman instrument for six times. All of the experiments were carried out at room temperature.

#### Characterization

The UV-visible spectra were recorded in a Shimadzu UV-2450 UV-visible spectrophotometer (Shimadzu Co. Ltd., Beijing, China) from 300 to 600 nm. DI water was used as the blank. SEM images were taken on a ZEISS-ULTRA 55 scanning electron microscope (Carl Zeiss AG, Oberkochen, Germany). For TEM, a drop of aqueous solution containing the samples was placed on the carbon-coated copper grids and dried under an infrared lamp for 30 min. The micrographs were obtained using a JEOL JEM-2010 transmission electron microscope (JEOL Ltd., Tokyo, Japan) operating at an accelerating voltage of 200 kV. Electron diffraction patterns were also recorded for the selected area. The surface charge of the samples was performed on NICOMP 380ZLS (Zeta potential/particle sizer; Agilent Technologies Inc., Santa Clara, CA, USA) system. SERS spectra of 2-Mpy-loaded AgMSs@GNPs were recorded by a simple Raman instrument (BWS415 B&W Tek Inc., Newark, DE, USA).

## Results and discussion

**Figure 2:**
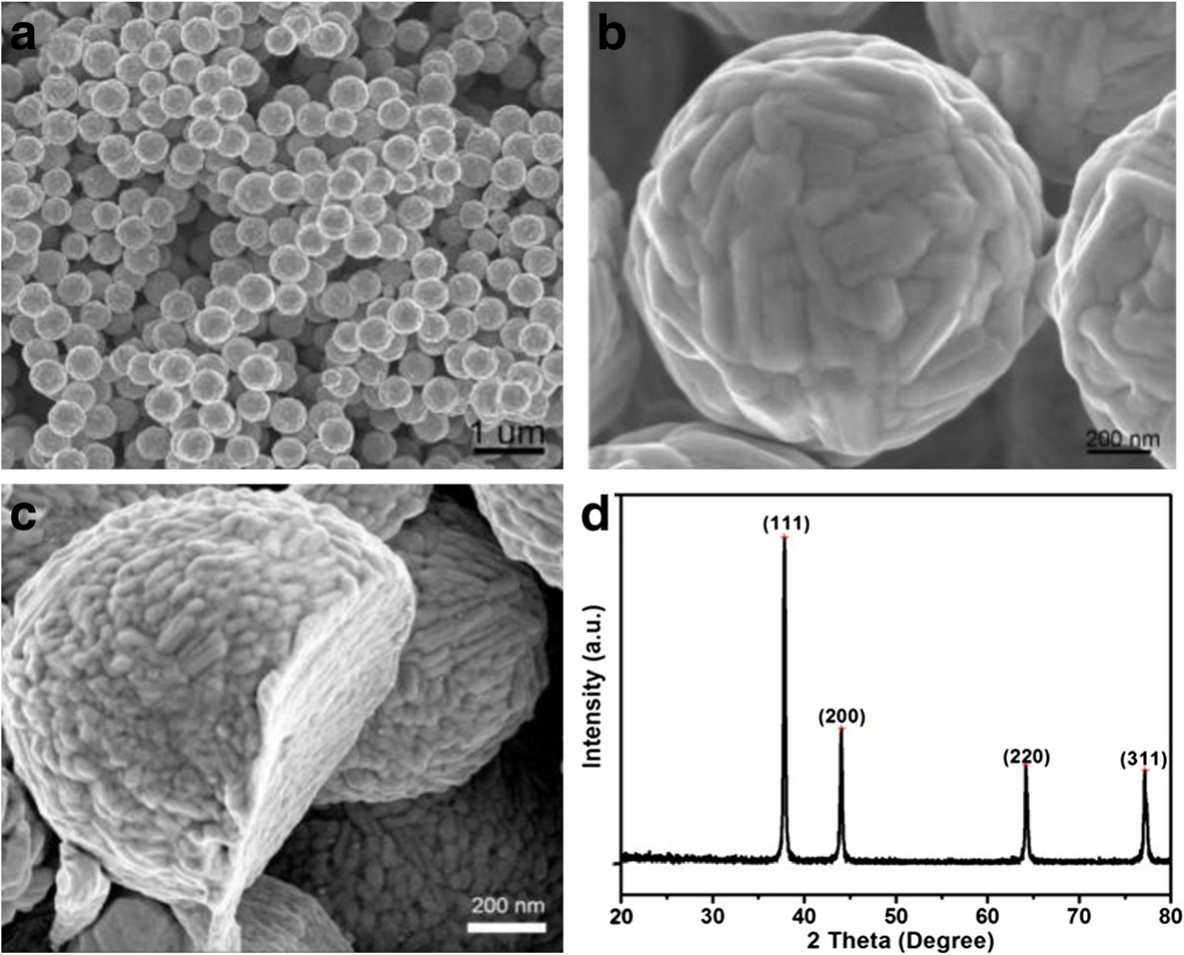
**SEM images of the AgMSs obtained from a typical experiment. (a)** Low-magnification SEM image of AgMSs, **(b)** high-magnification SEM image of an individual AgMS, **(c)** SEM image of an individual AgMS after cut by vibratome, and **(d)** XRD pattern of AgMSs.

**Figure 3:**
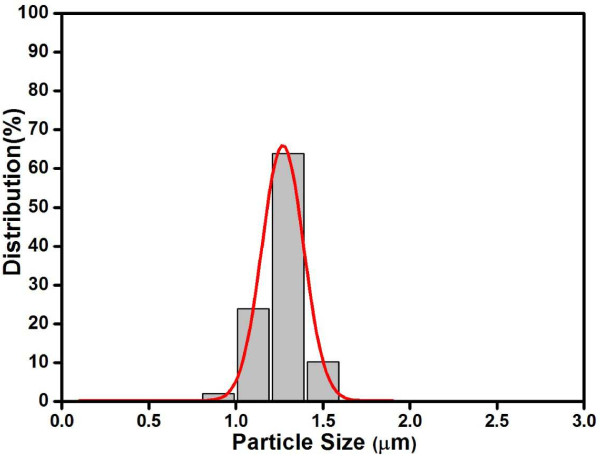
**Histogram showing the size distribution of Ag microspheres.** Gaussian curve is represented by a red line.

**Figure 4:**
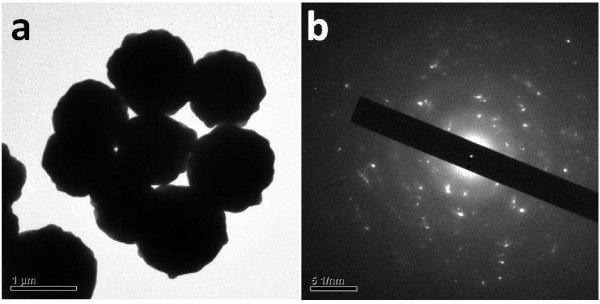
**TEM image and SAED pattern of Ag microspheres.** TEM image of Ag microspheres **(a)** and the selected area electron diffraction (SAED) pattern of the sample **(b)**.

In a typical synthesis of AgMSs, 2.5 mL of 5 mM aqueous solution of AgNO_3_ was added to 95 mL of deionized water in a 150 mL beaker. Then, 2.5 mL of 5 mM l-AA was added into the above-mentioned solution under vigorous stirring at room temperature. The system was stirred vigorously under ambient conditions for 4 h. During the whole process, there was no addition of any surfactants and/or organic solvents, and l-AA plays dual roles as both reducing and capping agent. Figure 2a shows the scanning electron microscopy (SEM) images of the AgMSs obtained from a typical experiment. The as-synthesized AgMSs are quasi-spherical with large quantity and good uniformity. The average overall diameter of Ag microspheres was 1.26 ± 0.11 μm, estimated by measuring 200 randomly selected spheres in the enlarged SEM images. The corresponding histogram of AgMSs shows the particle size distribution fitted by a Gaussian curve (Figure 3). The magnified SEM image (Figure 2b) indicates that these microspheres possess walnut-like rough morphologies with many trenches on their surfaces. To investigate the structure of AgMSs, the AgMSs were cut using a vibratome (UltraPro 5000; Leica Biosystems Inc., Weltzar, Germany) and observed by SEM, as shown in Figure 2c. It can be seen that the AgMSs are solid inside. Figure 2d is the X-ray diffraction (XRD) pattern of AgMSs. The peaks are assigned to diffractions from the (111), (200), (220), and (311) planes of face-centered cubic (fcc) Ag phase, respectively, which were in good agreement with the reference (JCPDS 04-0783). These planes with sharp peaks indicate that the AgMSs are all well crystallized. The peaks can be easily indexed to a pure cubic phase of silver. Meanwhile, no other impurity peaks were detected, suggesting the high purity of AgMSs. TEM is also performed to observe the morphologies of the as-prepared AgMSs (Figure 4a). The morphology of AgMSs is quasi-spherical, and the size is approximately 1.26 μm. There are some convex structures on the edges of microspheres, indicating that their surfaces are very rough. The results are consistent with the observation of SEM. The selected area electron diffraction (SAED) pattern (Figure 4b) further reveals that the AgMSs are crystalline with fcc structure and independent orientations.

**Figure 5:**
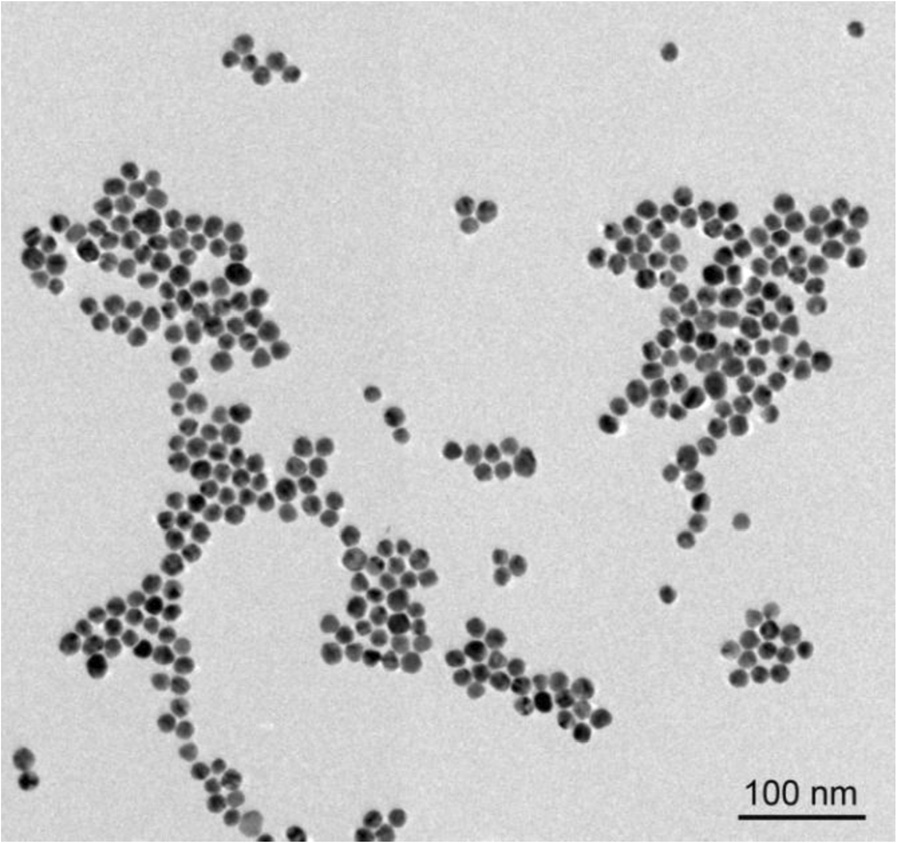
TEM image of gold nanoparticles dispersed in water.

**Figure 6:**
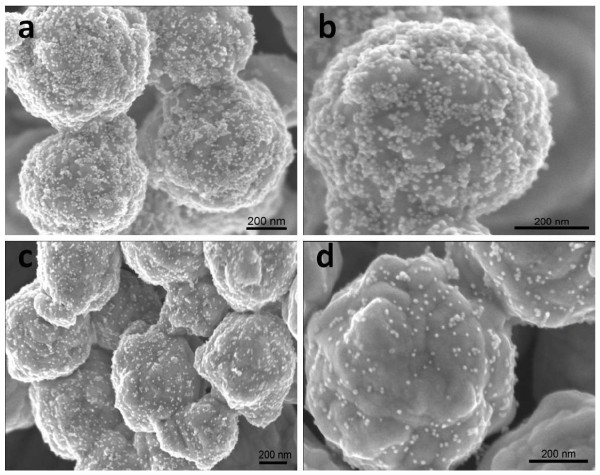
**SEM images of the assemblies prepared at molar ratios of AgMSs to GNPs. (a,b)** 100:20, **(c)** 100:2, and **(d)** 100:1.

Gold nanoparticles were synthesized according to our previous report [29]. TEM image of GNPs is shown in Figure 5, indicating that the GNPs are spherical and monodisperse with an average diameter of 15 nm. Based on the interaction between the carboxyl groups and silver atoms, the GNPs were successfully assembled on the surface of AgMSs [30]. Figure 6a,b,c,d clearly reveals that GNPs are homogeneously distributed on the surface of AgMSs. As can be seen, there are no changes in the shape and size of GNPs and AgMSs after self-assembly. With the increase of GNP concentration, the number of GNPs on the surface of AgMSs is also increased. When the molar ratio of AgMSs/GNPs is 100:20, the surface of AgMSs is completely coated by GNPs (Figure 6b).

**Figure 7:**
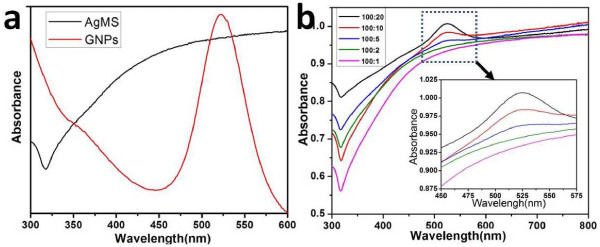
**Assemblies of AgMSs and GNPs detected by a UV–vis spectrophotometer. (a)** UV–vis spectra of AgMSs and GNPs; **(b)** UV–vis spectra of the assemblies prepared at different molar ratios of AgMSs to GNPs.

**Figure 8:**
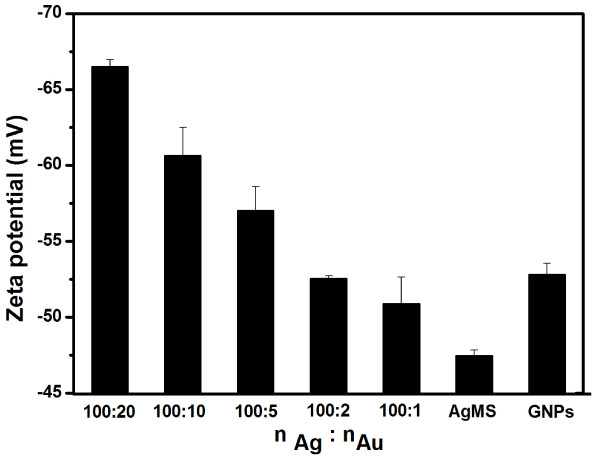
Zeta potential of the assemblies prepared at different molar ratios of Ag microspheres to gold nanoparticles.

To further testify the self-assembly between GNPs and AgMSs, the assemblies were also detected by a UV–vis spectrophotometer. As shown in Figure 7a, there is a strong absorption band in 350 to 600 nm for AgMSs. The broad half-peak width indicates that the size of AgMSs is bigger than nanoscale, which agrees with SEM and TEM observations. The absorption spectrum of GNPs displays a characteristic surface plasmon resonance band at approximately 520 nm. Figure 7b shows the UV–vis spectra of the assemblies prepared at different AgMSs/GNPs molar ratios. With the increase of GNP concentration, the intensity of the characteristic band at approximately 520 nm in the assemblies is also gradually increased. This is attributed to the increase of GNPs on the surface of AgMSs. The assemblies are negatively charged and display a GNP concentration-dependent increase of negative charges on the surface (Figure 8). The above facts suggest that the GNPs were successfully assembled on the surface of AgMSs.

**Figure 9:**
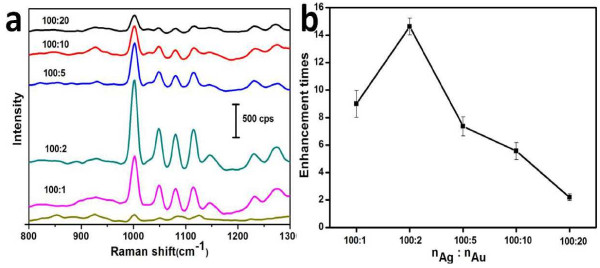
**SERS spectra of 2-Mpy molecules on the assembled substrates of AgMSs to GNPs. (a)** Representative SERS spectra of 2-Mpy (10^−7^ M) on the assembled substrates at different AgMSs to GNPs molar ratios. **(b)** The corresponding enhancement of the assembled substrates compared with 2-Mpy on pure AgMSs.

To evaluate the SERS activities of the assemblies of GNPs to AgMSs (AgMSs@GNPs), we chose 2-Mpy as targeted probing molecules [28]. Figure 9a shows the representative SERS spectra of 2-Mpy molecules on the assembled substrates of AgMSs to GNPs. All spectra exhibit peaks at 1,001, 1,049, 1,080, and 1,114 cm^−1^, which are assigned to the characteristic peaks of 2-Mpy molecules. Figure 9b shows the corresponding enhancement of the assembled substrates at different molar ratios of AgMSs to GNPs relative to 2-Mpy on pure AgMSs. Compared with the SERS activity of pure AgMSs, all AgMSs@GNPs exhibit obvious enhancement of SERS signal in varying degrees. The most significant enhancement of SERS signal is found at *n*_Ag_/*n*_Au_ ratio of 100:2, which is about 14-fold higher than that of pure AgMSs. Further increase of *n*_Ag_/*n*_Au_ ratio leads to decrease of SERS signal, which is likely due to the decreased nanogaps with increased gold particle deposition onto the surface of AgMSs. Several reasons can account for the enhanced Raman scattering signal: (1) The 3D assemblies of AgMSs@GNPs with huge, rough, and clean surface can absorb more molecules; (2) There are abundant ‘hotspots’ at the nanoparticles junctions to amplify the local E-fields as well as the Raman signal; and (3) AgMSs support the GNPs in 3D space to avoid the aggregation of the particles during application as SERS substrates.

## Conclusions

In summary, we report a simple, one-pot, surfactant-free synthesis of 3D AgMSs in aqueous phase at room temperature. The 3D AgMSs act as supports to fix the GNPs in 3D space via the interaction between the carboxyl groups of GNPs and the Ag atoms of AgMSs. The ensemble of AgMSs@GNPs with high SERS activity and sensitivity can be an ideal 3D substrate choice for practical SERS detection applications. The simple self-assembly strategy may be extended to other metallic materials with great potentials in SERS, catalysis, photoelectronic devices, etc.

## Competing interests

The authors declare that they have no competing interests.

## Authors’ contributions

SHC carried out the preparation of AuNPs, AgMSs, AgMSs@GNPs assembly, Raman and XRD, characterization, drafted the manuscript, PH modified the draft of manuscript, ZHW carried out the UV and SEM Characterization. ZW checked the manuscript grammar. MS participated in the analysis of Raman results. GN gave many advices for this manuscript. DXC and XYC designed of the study and guided this work. All authors read and approved the final manuscript.
